# A two-step algorithm for rapid diagnosis of active pulmonary tuberculosis in entry applicants using the T-SPOT.TB and Xpert MTB/RIF assays in Shanghai, China

**DOI:** 10.1038/emi.2017.52

**Published:** 2017-07-26

**Authors:** Chengyan Meng, Yaojie Shen, Jian Wang, Sen Wang, Xinchang Chen, Shenglei Yu, Wei Ye, Jing Wu

**Affiliations:** 1 Shanghai International Travel Healthcare Center, Shanghai 200335, China; 2 Department of Infectious Diseases, Huashan Hospital, Fudan University, Shanghai 200040, China; 3 State Key Laboratory of Genetic Engineering, School of Life Sciences, Fudan University, Shanghai 200433, China

**Dear Editor,**

With the development of the Chinese economy, the movement of people across the Chinese border has increased, thus, also potentially increasing the spread of tuberculosis (TB). Upon crossing the Chinese border, all entry applicants for work, education, or long-term settlement in Shanghai are required to undergo a medical examination, which includes a physical examination, chest radiography, and blood tests. If the radiograph indicates possible TB, conventional diagnostic methods are used for confirmation, which include acid-fast smear and *Mycobacterium tuberculosis* (MTB) cultures using sputum samples. Applicants with positive MTB cultures are diagnosed with TB; consequently, they are not cleared for entry and are required to leave China within one month. However, these applicants already waited 6–8 months to receive their results because of the limitations of conventional diagnostic methods (e.g., low sensitivity, long turnaround times and need for sophisticated laboratory facilities), which potentially increases the risk of TB transmission. Therefore, accurate and rapid detection of TB among entry applicants in China is necessary for timely treatment, reduced transmission and improved treatment outcomes of the disease.

Recently, novel diagnostic methods for TB have been introduced, including T-SPOT.TB (T-SPOT; Oxford Immunotec, UK) and Xpert MTB/RIF Assay (Xpert; Cepheid Inc, CA, USA). T-SPOT is an interferon-gamma release assay that uses blood samples to detect an immune response against MTB antigens. The test is not cross-reactive with Bacillus Calmette-Guerin or most non-tuberculous mycobacteria. The overall sensitivity of T-SPOT for diagnosing active TB is estimated to be 77%–88%.^1, 2, 3^ Xpert is an automated nucleic acid amplification test for the rapid detection of MTB DNA, usually from sputum samples. In 2010, the World Health Organization recommended Xpert as the initial diagnostic test for pulmonary TB for individuals at risk of multidrug-resistant or HIV-associated TB. Subsequently, Xpert was recommended as the initial diagnostic test for all individuals with suspected TB. Xpert is easy to use and has better sensitivity, fewer infrastructural requirements and faster turnaround times than conventional diagnostic methods. We conducted a study to analyze the diagnostic value of T-SPOT and Xpert for active TB among entry applicants in Shanghai and to determine an optimal algorithm for rapid and accurate TB diagnosis.

Our study was approved by the Shanghai International Travel Healthcare Center. The center is affiliated with the Shanghai Entry-Exit Inspection and Quarantine Bureau, which is responsible for the health administration of people traveling across the Chinese border in Shanghai. From January 2014 to December 2015, a total of 215 entry applicants had suspected TB based on chest radiography, and were required to undergo a sputum smear and culture tests. Sixty-eight of these applicants were willing to pay for a T-SPOT test; their sputum samples were also analyzed using Xpert free of charge. The volume of all sputum samples was adequate for smear, culture and Xpert tests. All 68 applicants who underwent sputum smear and culture, T-SPOT and Xpert tests were included in this study. The sputum was cultured on both a solid medium (Lowenstein−Jensen) and a liquid medium (BACTEC MGIT 960 culture). T-SPOT and Xpert were performed according to the manufacturer’s recommendations.

Of the 68 entry applicants, 23 were sputum culture-positive for MTB and were assigned to the TB group. Of these 23 active TB patients, 14 had negative smear microscopy results. In all, 45 individuals were sputum culture-negative for MTB. Of these 45 individuals, three had a positive smear microscopy and were culture-positive for non-tuberculous mycobacteria. These 45 individuals were monitored for three months for active TB symptoms. None of them developed active TB during the follow-up period, so we assigned these 45 people to non-TB group.

All T-SPOT results were interpretable. Of the 68, 47 persons (69.12%) were T-SPOT-positive and 21 of the 68 were T-SPOT-negative. All T-SPOT-negative individuals had negative sputum smear results. Compared to the sputum culture test, the sensitivity and specificity of T-SPOT were 100% and 46.7%, respectively. In Xpert, two samples returned an ‘invalid’ result. The other 66 samples had interpretable Xpert results, of which 20.59% (14/68) were positive and 76.47% (52/68) were negative. All Xpert-positive individuals were sputum culture-positive for MTB. Eight sputum culture-positive individuals (11.76%) were Xpert-negative. Seven (10.29%) acid-fast bacilli smear-negative individuals were Xpert-positive and sputum culture-positive for MTB. The overall sensitivity and specificity of Xpert were 65.2% and 100%, respectively. The sensitivity of Xpert was higher for smear-positive individuals than for smear-negative individuals (88.89% vs. 42.86%, *P*<0.05). The concordance rates of T-SPOT and Xpert compared with the sputum culture results were 64.71% and 88.24%, respectively.

For the rapid differentiation of TB from other diseases, we developed a two-step algorithm using T-SPOT followed by Xpert. We used T-SPOT as a rule-out test because it had the highest sensitivity. Because Xpert had the highest specificity, we used it as a rule-in test after initial screening with T-SPOT. If T-SPOT was used as the first step, 30.9% (21/68) of applicants with suspected TB could be correctly ruled out as not having TB within 48 h. If Xpert was used as the second step, 20.59% (14/68) of applicants with suspected TB could be correctly confirmed as having TB within only 90 min. If the two-step algorithm (T-SPOT test as a rule-out test followed by Xpert as a rule-in test) was applied, 51.47% (35/68) of applicants were correctly identified as having TB or not within 2 days, without requiring time-consuming sputum cultures. This drastically reduces the diagnosis time. The diagnostic flowchart is summarized in Figure 1.

Our data revealed that T-SPOT had 100% sensitivity for diagnosing TB among applicants with suspected TB. Because T-SPOT could not differentiate between latent TB and active TB,^4, 5, 6^ its specificity for active TB in areas with high TB prevalence is relatively low.^7^ Therefore, T-SPOT should only be used as a rule-out test for rapid diagnosis. The T-SPOT results indicated a high rate of MTB infection among applicants with suspicious findings on chest radiography, which was much higher than the average rate of latent TB.^8, 9^ T-SPOT-positive applicants need to be followed up with repeat chest radiography, sputum smear and culture tests every 1–2 months.

Our results of the Xpert for the diagnosis of TB are similar to those recently reported elsewhere.^10, 11, 12^ However, the sensitivity of Xpert in our study was similar to or lower than other studies, whereas its specificity was similar to other studies.^13, 14^ We also found that the sensitivity of Xpert for patients with smear-positive TB was higher than that for patients with smear-negative TB, similar to previous studies. In our study, we created a two-step algorithm for rapid diagnosis of TB at the Chinese border to prevent TB spread in China. By using the two-step algorithm, more than half of the applicants with suspected TB could be correctly confirmed as having TB or not within only 2 days.

Our study had a few limitations. First, the sample size was small; thus, future studies should include larger sample sizes. Second, Xpert had high specificity; however, some studies reported false-positive results,^15^ causing potential misdiagnosis.

In conclusion, Xpert is a promising and rapid method for diagnosing TB. Furthermore, using the two-step algorithm of T-SPOT and Xpert, more than half of the applicants were correctly identified as having TB or not within only 2 days. This approach will help with the rapid detection of TB, which allows for timely treatment, reduces transmission of the disease, and improves treatment outcomes.

## Figures and Tables

**Figure 1:**
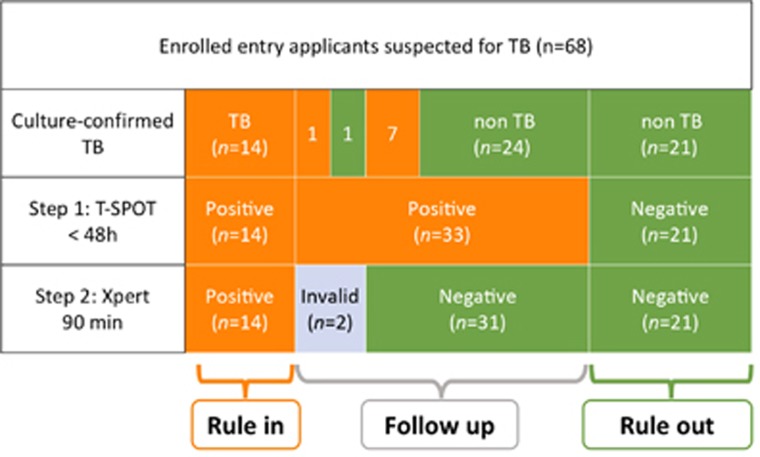
The diagnostic flowchart. Of the 68 entry applicants, 23 were sputum culture-positive for MTB and were confirmed as having TB; 45 individuals were sputum culture-negative for MTB and were confirmed as not having TB. A two-step algorithm using T-SPOT followed by Xpert could be used for the rapid diagnosis of TB. Using T-SPOT as Step 1, 47 of 68 applicants were T-SPOT positive and 21 of 68 applicants were T-SPOT-negative. Using Xpert as Step 2, 14 of 68 applicants were Xpert-positive and 52 of 68 applicants were Xpert-negative, with two ‘invalid’ results. For the rapid diagnosis of TB, T-SPOT can be used as a rule-out test followed by Xpert as a rule-in test. T-SPOT-positive, Xpert-negative patients need to be followed up with repeat chest radiography, sputum smear and culture tests every 1–2 months. *Orange:* individuals with positive results for the indicated test; *Green:* individuals with negative results for the indicated test.
